# Indicators to Examine Quality of Large Scale Survey Data: An Example through District Level Household and Facility Survey

**DOI:** 10.1371/journal.pone.0090113

**Published:** 2014-03-05

**Authors:** Kakoli Borkotoky, Sayeed Unisa

**Affiliations:** 1 International Institute for Population Sciences (IIPS), Mumbai, India; 2 Department of Mathematical Demography and Statistics, IIPS, Mumbai, India; Tulane University School of Public Health and Tropical Medicine, United States of America

## Abstract

**Background:**

Large scale surveys are the main source of data pertaining to all the social and demographic indicators, hence its quality is also of great concern. In this paper, we discuss the indicators used to examine the quality of data. We focus on age misreporting, incompleteness and inconsistency of information; and skipping of questions on reproductive and sexual health related issues. In order to observe the practical consequences of errors in a survey; the District Level Household and Facility Survey (DLHS-3) is used as an example dataset.

**Methods:**

Whipple's and Myer's indices are used to identify age misreporting. Age displacements are identified by estimating downward and upward transfers for women from bordering age groups of the eligible age range. Skipping pattern is examined by recording the responses to the questions which precede the sections on birth history, immunization, and reproductive and sexual health.

**Results:**

The study observed errors in age reporting, in all the states, but the extent of misreporting differs by state and individual characteristics. Illiteracy, rural residence and poor economic condition are the major factors that lead to age misreporting. Female were excluded from the eligible age group, to reduce the duration of interview. The study further observed that respondents tend to skip questions on HIV/RTI and other questions which follow a set of questions.

**Conclusion:**

The study concludes that age misreporting, inconsistency and incomplete response are three sources of error that need to be considered carefully before drawing conclusions from any survey. DLHS-3 also suffers from age misreporting, particularly for female in the reproductive ages. In view of the coverage of the survey, it may not be possible to control age misreporting completely, but some extra effort to probe a better answer may help in improving the quality of data in the survey.

## Introduction

Sample surveys are an important source of the data on various demographic and health related indicators of a country. Maintaining good quality of data thus becomes the fundamental objective in any survey. Therefore, quality checks are also required so that these surveys produce high quality data that give a true representation of the economic, social and demographic indicators of the country. Evaluation of the quality of data is important in order to ensure the accuracy of the conclusions drawn from the data. In India, different cross sectional surveys are conducted to capture the changes in the country's socio-economic and demographic indicators on a regular basis. Quality of data is affected by both sampling and non-sampling errors. Among non-sampling errors; respondents under-reporting of events, incorrect recording of information by interviewer, errors arising from questionnaire design etc. are of more serious nature. Error due to non-response arises when some units do not respond or not investigated at all. In order to improve the accuracy and reliability of sample surveys, it is necessary to minimize both sampling and non-sampling errors. Some other potential sources of error may be, variability in response, bias and variation arising from the interviewer, and due to the faulty selection of date or period of the survey [1].

Among different sources of sampling and non-sampling errors, the most commonly encountered error in a census or survey is inaccurate age reporting. It has been said that data collected through census or sample surveys in developing countries are more likely to have irregularities in age reporting than data collected in developed countries [2]. Further, it has been said that age misreporting may occur due to ignorance of actual ages, miscommunication between interviewers and respondent, in order to meet social norms regarding the relationship of age to other social characteristics or due to errors during recording or processing [3]. Some other kinds of errors that are observed in demographic surveys are the systematic transfer of respondents from the border of an eligible age group to the neighbouring group to avoid individual interviews [4], [5]. Such under-enumeration not only affects fertility related indicators but also affect the age structure of the population. Another possible source of error in age reporting may be misreporting of age at first marriage in an attempt to hide low age at marriage. In another possibility, interviewer may change the birth year of children to avoid asking questions on immunization and other health related issues [5].

Different studies have been conducted [6], [7], [8] to examine the quality of data for census and other surveys like National Family Health Survey (NFHS). All these results support that information gathered from uneducated respondents is more erroneous than that from educated groups. Out of all the possible sources of errors in surveys, age misreporting is the most challenging one [9]. In countries where a large section of the population is illiterate, age reporting in the census and surveys are likely to be inaccurate, and subsequently, the errors in reported ages would be transmitted to estimates based on them [10]. In particular, if women's ages are misstated, even an accurate enumeration of the total births will result in distortions, in age-specific fertility rates [11]. In developing countries like India, where a large portion of the population is illiterate, the age returns from the censuses and surveys suffer from misstatement on account of ignorance of age, deliberate misstatement and misunderstanding of the question [12].

In addition to the errors arising from age misstatement, another factor that affects data quality is; skipping of questions in order to avoid answering few sections in the questionnaire. This error may creep in either due to the respondent or the interviewer. Single or multiple items may be missing because the respondent unconsciously skips an item or block of items or refuses to answer the questions. Sometimes, the respondent may not have the information to answer the question, and this may occur more frequently when the respondent is a proxy for another person [13]. Another possibility may be that, the interviewers may not ask questions properly or follow directions for skip exactly, either purposefully or because the directions have not been made clear enough. All these will lead to missing information. In some cases, a poor design of the questionnaire may confuse respondents, leading to a misunderstanding of skip patterns [14]. Therefore, it is important that, the directions for skip should be followed properly so that any relevant information is not missed from the survey.

On the basis of the above discussions, in this paper we examined three categories of possible errors that may occur in any survey; age misreporting, incompleteness, and inconsistency of information. In order to examine the practical consequences of all these errors on a large scale survey, DLHS-3 data have been used. Although there have been concern regarding data from different surveys conducted in India, but there is lack of studies that focus on the quality of data from District Level Household Survey (DLHS-3), which is another important source of demographic data in India. The quality of data in large scale surveys like DLHS has significance because it is the only large scale survey that provides information on social and demographic indicators up to the district level in India, and the estimates are also used in formulating policies. In this paper, importance has been given to age data since the effect of age misreporting will be visible through deformation of the age structure of the population and imbalanced sex ratio. Age misreporting could also affect the estimation of several vital events, including an increase in the frequency of events for a particular period in the past. The quality of data has been examined for different background characteristics of the respondents. In addition to age related information, we also examined the association of fieldwork related factors with the quality of data. Further, we examined the response to the questions on age at marriage, number of pregnancies during the reference period, pregnancy status at the time of the survey, Ante Natal Visits, birth year of children, and knowledge about Human Immune deficiency Virus (HIV) and Reproductive Tract Infection (RTI) to examine the consistency and completeness of response.

## Data Source and Methodology

### Source of Data

The study used data from the District Level Household and Facility Survey-3 (DLHS-3) [15] conducted during 2007–08 as an example dataset to show, how quality of data may be affected by misreporting of ages, and lack of consistency and completeness of response. In order to compare the changes in the pattern of age reporting, the study also used DLHS-2 (2002–04) data. The DLHS is one of the largest ever demographic and health surveys carried out in India, which is designed to provide estimates at the district level. The National Family Health Survey (NFHS), which is the Indian version of Demographic and Health Survey; gives information up to the state level. In order to go beyond NFHS and overcome the difficulties of getting information on district level indicators, DLHS survey was introduced. The DLHS-3 was carried out in 601 districts of India covering 7,20,320 households from 34 states and union territories of India. The earlier rounds of District Level Household and Facility Survey were carried out in 1998–99 (DLHS-1) and 2002–04 (DLHS-2). The DLHS-3 aims at providing estimates on maternal and child health, family planning and other reproductive health indicators at the district level. The DLHS-3 survey interviewed both married (15–49), and unmarried (15–24) women. The survey used separate questionnaire to collect information from household, ever-married women, unmarried women, village and health facility. Given the objectives of the DLHS-3 survey, reporting the true age for female during household interview is important so that women are not excluded from the survey.

### Ethics Statement

The District Level Household and Facility Survey (DLHS-3) was conducted by the International Institute for Population Sciences (IIPS), Mumbai, India. The survey obtained informed consent from the respondents who agreed to participate in the study. The sources of funds for DLHS-3 are the Ministry of Health and Family Welfare, Government of India; United Nations Population Fund (UNFPA) and United Nations Children's Fund (UNICEF). This study is based on DLHS-3 data, which is available on public domain with all the identifiable information removed from the data.

### Methodology

We examined the quality of age related information collected through household and individual questionnaire from respondents with different background characteristics. Household questionnaire in DLHS-3 contains information on all regular members of the household and visitors. The individual questionnaire includes sections on respondent's characteristics, ante-natal, natal and post-natal care, immunization and child care, knowledge and use of contraception and reproductive health. Individual interviews are conducted with women in 15–49 age group listed in the household questionnaire. Age reporting during household interview thus becomes important, since respondents for individual interview are selected from household interview. In order to evaluate the quality of data used to determine eligibility for individual questionnaire, we examined age reporting in the household questionnaire to identify exclusion of women from the individual questionnaire resulting from misreporting of age.

For measuring the age misreporting or digit preference, we used Whipple's and Myers indices. The values of Whipple's index were classified into three categories as, low (100–150), moderate (150–250) and high (>250) to identify digit preference based on the range. The following formula was applied to calculate Whipple's index [16]

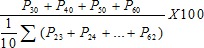
Another widely used index of digit preference is Myer's index. This is a summary index of preference of all terminal digits derived as one half of the sum of the deviations from 10.0 percent. The range of Myers index is 0 to 90. The range is further classified into three categories as, low (<10), moderate (10–20) and high (>20).

### Age Boundary effects

The misreporting of women's age can lead to their exclusion from the individual interview by pushing them out of the eligible age range [17]. It may occur due to both interviewer bias and respondent bias. Interviewers may push women out of the eligible age range in order to reduce the number of women to be interviewed. Also, the household informant may exclude some eligible women from being interviewed for personal reasons. There is a higher probability of misreporting the ages of women who are near the upper and lower limits of eligible range. In order to summarize the extent of distortion in the age/sex structure of the sample near the age eligibility boundaries, indices are calculated based on age and sex ratios. Three indices are considered

**L** (lower boundary distortion), **U** (upper boundary distortion) and **T** (total of upper and lower boundary distortion). The L and U indices are defined as
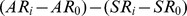
Where AR and SR are the age ratios and sex ratios, subscript ‘i’ denote the age group inside the boundary (i.e. 15–19 and 45–49) and subscript ‘o’ denotes the age group outside the boundary (i.e. 10–14 and 50–54). A positive sign indicates that too many women were considered eligible (in transference), and a negative sign indicates that too many were considered ineligible (out-transference). The index for total boundary distortion, T is calculated as
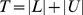
For calculating the index T, sign of L and U are disregarded, because the movement of women at one boundary can be offset by movement at the other boundary. Hence, the T index indicates only the degree of distortion, not its direction. The range for this index is defined as, Negligible (0–24), Low (25–49), Moderate (50–99) and High (100+).

To examine the proportion of female transferred downward or upward, we used a method widely used to examine the age displacement of women [5], [18]. The method uses four successive age intervals of equal width (single years or five year age groups), two of which come before the boundary and two of which come afterwards. The age groups used in this study, to estimate the downward and upward shift of women from the DLHS-3 survey is given in Table 1.

**Table 1:** The age groups used to estimate downward and upward shift of women from eligible age group (15–49) for DLHS-3 survey.

Age groups for downward shift	Age groups for upward shift	Observed Frequency	Fitted Frequency
5–9	40–44	a	a
10–14	45–49	b	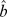
15–19	50–54	c	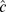
20–24	55–59	d	d

The development of the model is based on two assumptions:

First, 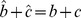
Second, based on the assumption that changes in both cohort size and force of mortality tend to be linear on a log scale, the ratios 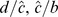 and 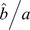 are also linear on log scale. Given the second assumption, the following equation is derived -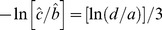
Where, 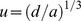, 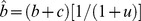, and 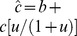


Then to estimate the percentage of downward and upward shift by misreporting of ages, the formula is given:
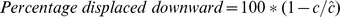


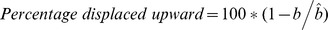
In addition to examining the pattern of age reporting, we also examined the consistency of information during the interview. We examined the consistency of data with the help of five important questions; number of pregnancies in the five years prior to survey, any Ante Natal Care (ANC) visit, current pregnancy status, and knowledge about HIV and Reproductive Tract Infection (RTI). These questions have been selected because; moving forward to continue the interview to collect information on the number of ANCs, complications and care during pregnancy, knowledge about the spread of HIV and RTI will depend on whether the respondent had any pregnancies during the reference period and whether she had ever heard about HIV or RTI. Skipping these questions will lead to lack of information on birth history, ante-natal, natal & post-natal care, and reproductive & sexual health related issues. We tried to examine whether the response to these questions is consistent with the earlier responses of the respondent.

## Results

### Fieldwork Related Factors and quality of data

In our study, we considered the number of visits to the household and timing of visit as two indicators for fieldwork related factors. The percentage of one or more visits to households and the time of visit for data collection is presented in Table 2. Number of visits to the household to collect data may be associated with the effort of collecting good quality data. Here, the range of variation among the states, in case of one visit is very low. Majority of the states reported only a single visit to the household for collecting data. This finding may give rise to the question, whether the interviewers could complete more than 90 percent of the interviews through a single visit only, in all states. Only in six states, including four southern states, more than one visit have been reported, with the frequency of more than one visit ranging from 10–17 percent. The response to an interview also depends on the time of visit. Most of interviews for the survey are conducted in the morning time. During this time mostly female members are present in the household. Also, they remain busy with their household activities, so the response obtained may not be accurate. It will be reflected in the overall response rate. In an attempt to capture wrong reporting of the time of an interview, the study classified midnight to early morning as odd time. Interestingly, some of the interviews fall in this range, as well. It may be either due to interviewer bias or may be the result of wrong data entry.

**Table 2:** Number and Time of visit to collect information from the household during the DLHS-3 survey.

State	Number of Visits		Time of Visit			
	One visit	Two or more visits	Morning	Afternoon	Evening	Odd time
Andhra Pradesh	89.5	10.6	70.7	26.9	2.1	0.3
Arunachal Pradesh	98.4	1.6	71.2	27.5	0.8	0.5
Assam	97.1	2.9	61.8	37.3	0.7	0.1
Bihar	96.8	3.2	48.7	49.7	1.5	0.1
Chhattisgarh	89.7	10.3	55.9	41.2	2.9	0.1
Delhi	90.1	9.9	51.2	45.7	3.1	0.04
Goa	84.0	15.9	47.2	40.8	11.8	0.1
Gujarat	93.4	6.6	58.7	38.1	2.9	0.4
Haryana	96.8	3.2	64.6	34.0	1.4	0.03
Himachal Pradesh	95.6	4.4	58.2	39.3	2.4	0.04
Jammu & Kashmir	94.9	5.2	55.5	42.9	1.5	0.2
Jharkhand	96.2	3.8	61.8	37.6	0.5	0.1
Karnataka	87.6	12.4	49.8	36.2	13.8	0.2
Kerala	82.7	17.3	61.9	37.9	0.1	0.2
Madhya Pradesh	91.7	8.3	54.1	43.9	1.9	0.1
Maharashtra	91.8	8.2	53.2	41.7	4.9	0.2
Manipur	93.8	6.2	65.8	31.3	2.1	0.7
Meghalaya	95.1	4.9	48.9	48.9	2.1	0.1
Mizoram	94.4	5.6	60.6	34.5	4.5	0.4
Orissa	91.7	8.3	63.8	35.2	0.7	0.3
Punjab	97.1	2.9	76.9	22.4	0.5	0.1
Rajasthan	96.1	3.9	62.2	36.6	1.1	0.1
Sikkim	95.6	4.4	52.1	45.3	2.5	0.1
Tamil Nadu	84.7	15.3	73.8	22.3	3.7	0.2
Tripura	97.0	2.9	60.1	39.2	0.6	0.1
Uttar Pradesh	96.7	3.3	58.0	40.9	1.1	0.1
Uttarakhand	95.2	4.9	53.4	39.4	7.0	0.2
West Bengal	95.8	4.2	61.5	37.8	0.6	0.0

### Respondent Characteristics and its association with Age Data

In order to examine the variations in age reporting from respondents with different socio-economic background; indices of digit preference are calculated by respondent characteristics and the results are presented in Table 3. The results show that digit preference in age reporting is higher among male than female. When we consider education of the respondent, the Myer's index is high for illiterate than literate. It is also evident from the Whipple's index for the states, where Kerala being the most literate state, has the lowest value of the index. If we take into consideration, the religion of the respondent, age misreporting is present in every religion but, highest misreporting is noticed among Muslims as compared to other religions. Locality of the household is considered as an important factor in age reporting. In general, it is assumed that, age reporting will be better in urban areas than rural areas. The results also confirm the general belief because, the value of Myer's index is higher for rural areas and people belonging to the poorest wealth quintile. The value of the index improves with improvement in the economic condition. The values obtained for different wealth quintile also show that poor people have a higher tendency of misreporting their ages. Among all these factors, the most significant difference in age reporting has been observed for literate and illiterate respondents.

**Table 3:** Age misreporting in DLHS-3 survey measured by Myers Index according to respondent characteristics.

Background Characteristics	Myers Index
**Place of residence**	
Rural	23.8
Urban	18.6
**Sex**	
Male	25.3
Female	19.8
**Literacy**	
Literate	18.0
Illiterate	31.3
**Religion**	
Hindu	23.0
Muslim	23.2
Christian	16.8
Sikh	22.7
**Wealth Quintile Index**	
Poorest	28.5
Second	25.9
Middle	22.9
Fourth	20.4
Richest	16.9

### Pattern of age reporting at the state level

**Figure 1:**
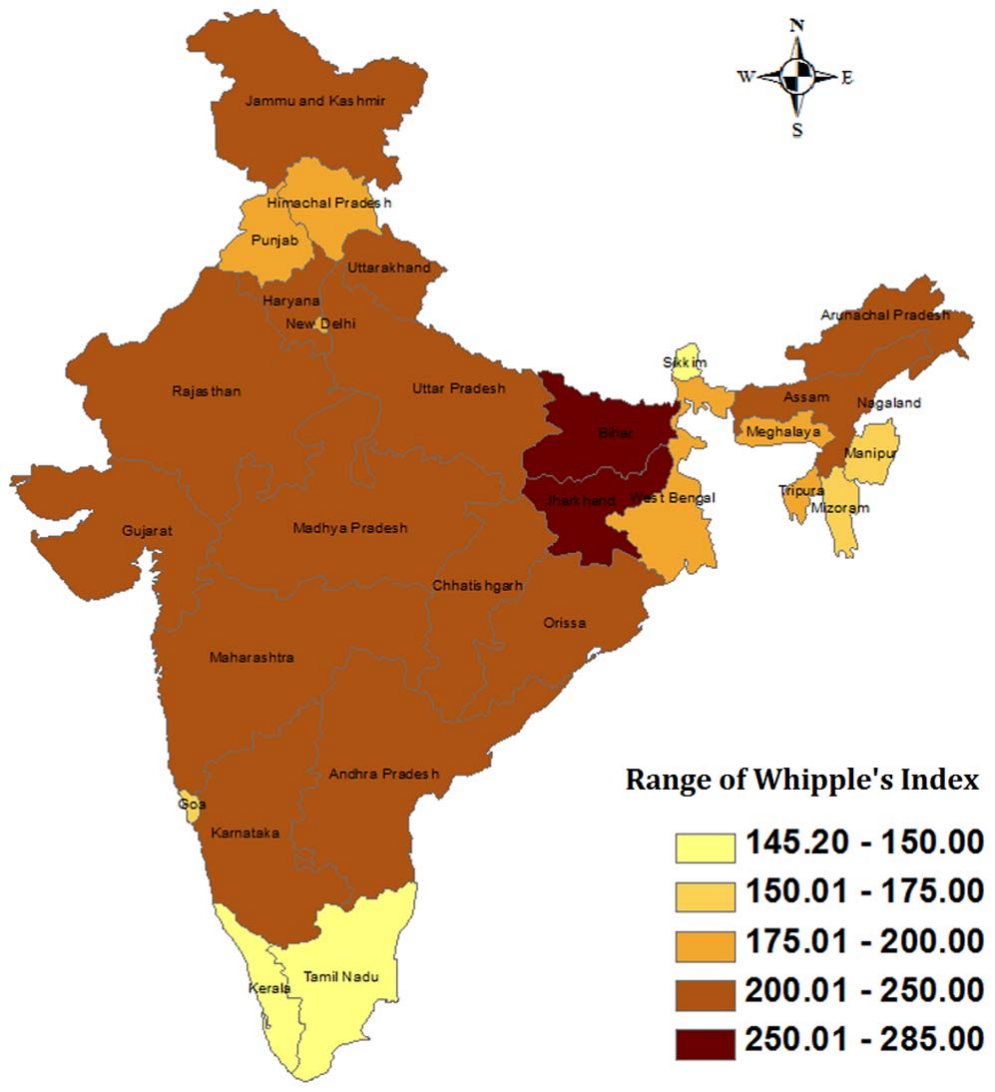
State wise pattern of age reporting in DLHS-3 measured by Whipple's Index.

**Figure 2:**
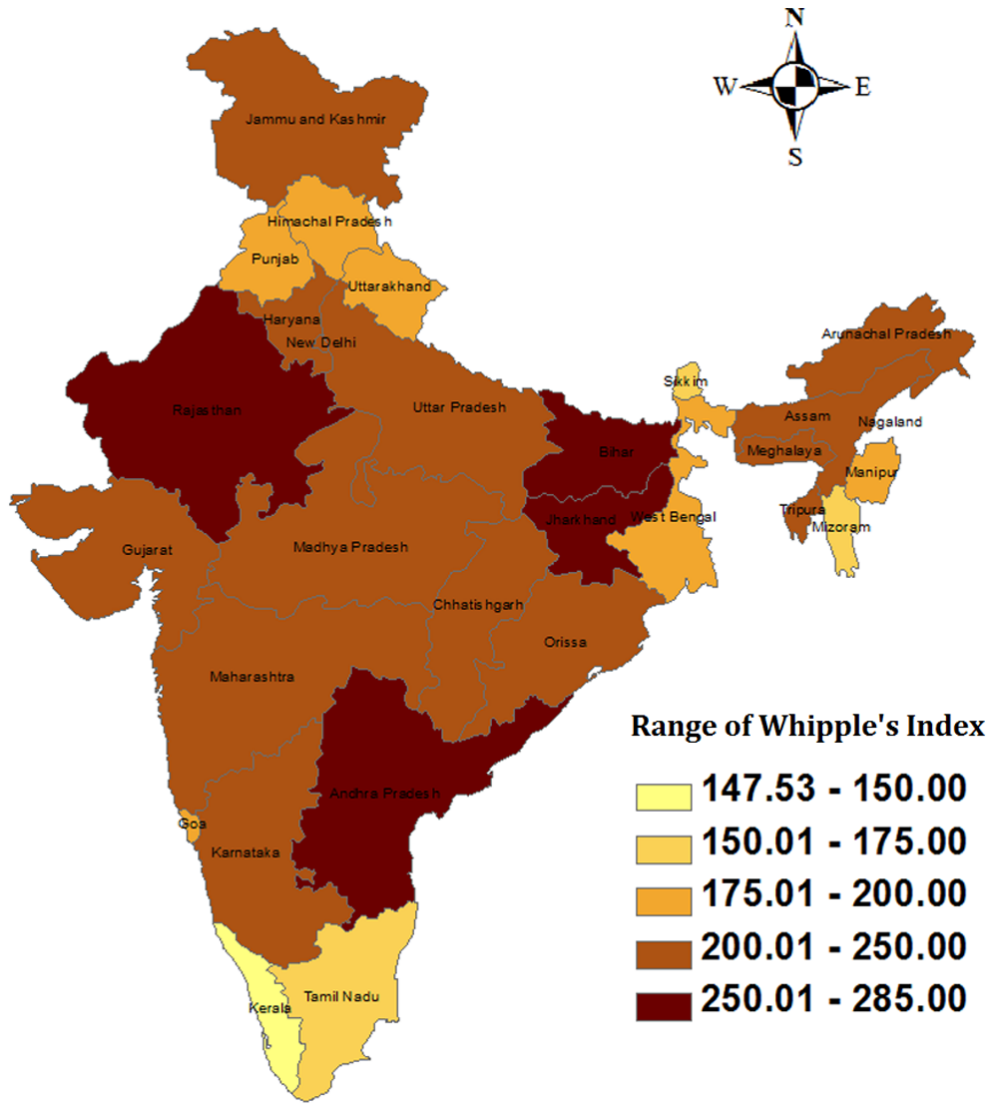
State wise pattern of age reporting in DLHS-2 measured by Whipple's Index.

We examined the pattern of age reporting at the state level for the two rounds of DLHS (DLHS-2 and DLHS-3) survey with the help of Whipple's index, and the results are presented in Figure 1 and Figure 2 respectively. The states are divided into five groups on the basis of the values of Whipple's index, and the classification is presented in the legend. On the basis of the comparison, we may say that, on the whole, age reporting has improved from DLHS-2 to DLHS-3. The figure shows improvement in the level of age reporting for the states which had very high levels of age misreporting. In DLHS-2 four major states viz. Bihar, Jharkhand, Rajasthan and Andhra Pradesh had high digit preference in age reporting, but, Rajasthan and Andhra Pradesh showed improvement in age reporting during DLHS-3. However, majority of the states fall in the moderate range of age misreporting in both the rounds of the survey. Only two southern states viz. Kerala and Tamil Nadu had lower levels of age misreporting in both the rounds of the survey. This implies that special care should be taken while collecting age related information during survey.

### Age misreporting and its impact on age and sex ratio of adjacent age groups

Age and sex ratios for the four age groups 10–14, 15–19, 45–49, and 50–54 are examined, and the results are presented in Table 4. Importance is given to these age groups because women in the 15–49 year age group are eligible for individual interview, and misreporting of age in this age range will influence the results of the survey to a greater extent. If the ages of young women were systematically understated in order to avoid eligibility, the age ratio for the 15–19 age group would be low and sex ratio will be high in compared to the 10–14 age group. The opposite will be true if more than the actual number of women is included in the 15–19 age group. The ratios are presented separately for each state, to examine in- transference and out-transference of women from the eligible age range.

**Table 4:** Degree of age displacement for women in the lower and upper limits of reproductive age group in the DLHS-3 survey.

State	Age Ratio				Sex Ratios				L^1^	U^2^	T = |L|+|U|
	10–14	15–19	45–49	50–54	10–14	15–19	45–49	50–54	15–19	45–49	
Andhra Pradesh	105.7	92.7	83.9	119.5	96.5	102.9	124.1	77.5	−19.5	−29.8	49.4
Arunachal Pradesh	113.2	106.9	120.7	124.6	118.6	137.5	102.1	108.6	−25.4	2.6	27.9
Assam	119.9	81.9	62.9	196.1	96.8	120.5	167.9	60.1	−61.7	−66.5	128.2
Bihar	101.0	91.6	91.2	90.4	106.6	94.9	113.9	96.9	2.2	−16.2	18.4
Chhattisgarh	104.9	97.6	92.2	84.7	97.2	101.2	142.1	100.5	−11.3	−46.4	57.7
Delhi	110.2	91.7	78.7	135.5	114.8	139.2	146.6	99.4	−42.9	−104.0	146.9
Goa	96.8	102.1	96.6	108.3	97.4	91.2	99.2	84.2	11.5	−13.5	25.0
Gujarat	107.1	92.2	78.9	131.1	106.9	111.1	130.7	80.9	−18.9	−32.9	51.9
Haryana	108.8	96.6	116.2	70.5	114.4	122.2	113.9	140.3	−20.0	72.1	92.2
Himachal Pradesh	118.0	93.2	65.7	158.4	99.0	118.7	156.8	71.3	−44.6	−178.2	222.7
Jammu & Kashmir	109.1	101.0	80.9	125.2	100.1	114.5	136.2	85.4	−22.4	−95.0	117.4
Jharkhand	120.4	77.7	48.3	213.7	98.5	128.1	190.7	49.9	−72.4	−91.9	164.3
Karnataka	98.9	104.4	94.6	100.6	101.9	95.9	126.3	101.4	11.4	−40.1	51.6
Kerala	106.6	89.8	90.0	115.2	102.4	104.5	103.1	79.5	−18.9	1.6	20.5
Madhya Pradesh	105.6	95.8	92.4	109.1	107.0	111.5	121.5	95.8	−14.4	−13.4	27.7
Maharashtra	107.8	100.3	83.8	104.2	100.9	110.0	132.1	97.5	−16.7	−38.5	55.2
Manipur	108.9	90.6	77.0	163.0	102.2	111.7	119.2	72.4	−27.9	−132.8	160.7
Meghalaya	123.9	85.3	71.5	215.2	94.8	130.5	136.9	63.6	−74.2	−217.1	291.3
Mizoram	96.5	98.8	92.8	139.6	106.9	115.5	108.9	91.0	−6.3	−64.7	71.1
Orissa	112.6	89.4	73.2	146.7	95.8	96.9	137.8	72.2	−24.3	−57.1	81.3
Punjab	105.2	99.0	100.3	87.9	117.9	123.3	120.9	106.1	−11.7	−2.5	14.2
Rajasthan	108.0	98.6	94.5	91.6	113.7	116.2	128.5	102.9	−11.9	−22.7	34.6
Sikkim	118.9	104.8	94.1	126.5	100.2	105.4	119.6	117.5	−19.3	−34.5	53.8
Tamil Nadu	99.5	93.2	92.9	101.3	108.9	101.5	109.5	80.0	1.0	−8.2	9.2
Tripura	94.4	105.8	77.7	130.7	102.2	98.4	152.7	79.1	15.1	−126.6	141.7
Uttar Pradesh	106.9	101.3	95.4	83.4	107.8	106.9	124.8	107.9	−4.7	−4.9	9.6
Uttarakhand	117.1	94.2	53.2	184.1	100.4	120.7	180.6	58.7	−43.2	−252.9	296.1
West Bengal	91.5	114.7	98.9	95.2	111.4	87.6	122.6	110.9	47.1	−50.8	97.9

1 lower boundary distortion.

2 upper boundary distortion.

The results on distortion of age reporting show that, in most of the states, female were excluded from the lower limit of eligibility i.e. 15–19 age group. Only in four states viz. West Bengal, Tripura, Karnataka, and Goa, age ratio for 15–19 is higher than the age ratio for 10–14 age group, which means that, in these states more than the actual number of female was included in 15–19 age group. It is also important to mention that the highest age ratio in 10–14 age group is obtained for Meghalaya, representing that the displacement of women from the 15–19 age group is highest in this state. This result bears significance because Meghalaya has one of the highest literacy rates in the country and thus, high level of age misreporting is not expected in this state. The sex ratio in 15–19 age group is highest in Jharkhand (190) followed by Arunachal Pradesh (137), Meghalaya (130) and Uttarakhand (120). For these states, age ratio in 15–19 was lower than the age ratio in 10–14. Lowest sex ratio in 15–19 age group is observed for West Bengal (87), this is resulting from inclusion of more female in 15–19 age group as observed in the table. Similarly, results obtained for age and sex ratios in the 45–49 and 50–54 age group imply that the survey suffer from out transference of eligible women for the upper age group, as well. The results indicate that misreporting of women's age is more or less common in every state, only the extent of misreporting may vary. The classification of the states in the four categories on the basis of the index (|T|) has been summarised in the Table 5. It can be clearly observed that, on the basis of degree of age displacement, only five states come under the negligible category and four others in the category of low age displacement.

**Table 5:** Classification of the states based on degree of age displacement for women in the DLHS-3 survey.

Negligible (0–24)	Low (25–49)	Moderate (50–99)	High (100+)
Bihar	Arunachal Pradesh	Andhra Pradesh	Assam
Kerala	Goa	Chhattisgarh	Delhi
Punjab	Madhya Pradesh	Gujarat	Himachal Pradesh
Tamil Nadu	Rajasthan	Haryana	Jammu & Kashmir
Uttar Pradesh		Karnataka	Jharkhand
		Maharashtra	Manipur
		Mizoram	Meghalaya
		Orissa	Tripura
		Sikkim	Uttarakhand
		West Bengal	

### Percentage of Women Displaced from Eligible Age Group

Age Displacement of women may have consequences on the measures produced by the data. Therefore, it is important to know the percentage of women that have been displaced from the lower (15–19) and upper (45–49) limits of eligible age group. The results obtained by comparing the observed and fitted frequency for these two age groups are presented in Table 6. The results show that a high percentage of female was excluded from the upper age boundary in Jharkhand (45 percent), Uttarakhand (38 percent), Assam (34 percent), and Meghalaya (33 percent). In comparison to the upper age limit, less variation in age reporting has been observed for the lower age group. Even then the overall impact of the changes in the age group will be visible in the indicators. In case of lower age boundary, 16 states shows exclusion of female, and for the upper age boundary 21 states show exclusion of female from the actual age group. Age Displacement are most likely due to intentional efforts by interviewers to reduce their workload because the respondents themselves may not be aware of the screening function and have no incentive to shift ages across the boundaries [13]. This kind of errors is very difficult to identify once the data collection and entry are completed.

**Table 6:** Percentage of Female displaced from the lower and upper limit of reproductive age group in the major states of India during DLHS-3 survey.

State	Observed frequency		Fitted frequency		% of female displaced	
	15–19	45–49	15–19	45–49	15–19	45–49
Andhra Pradesh	5926	2498	5986.5	2816.7	1.0	11.3
Arunachal Pradesh	5368	2769	5586.3	2853.8	3.9	2.9
Assam	11629	3098	11600.6	4712.2	−0.2	34.3
Bihar	17632	4560	18441.6	4530.1	4.4	−0.7
Chhattisgarh	5704	1942	5799.4	1877.8	1.7	−3.4
Delhi	2346	940	2263.3	1125.7	−3.7	16.5
Goa	378	259	388.4	269.1	2.7	3.8
Gujarat	7273	2955	7335.8	3506.7	0.9	15.7
Haryana	6274	2575	6061.5	2225.8	−3.5	−15.7
Himachal Pradesh	3290	1355	3147.2	1862.4	−4.5	27.3
Jammu & Kashmir	5547	2013	6196.7	2329.6	−0.3	13.6
Jharkhand	11851	2134	11466.9	3883.1	−3.4	45.0
Karnataka	7720	3381	7763.6	3442.7	0.6	1.8
Kerala	2843	2003	2755.7	2174.2	−3.2	7.0
Madhya Pradesh	16753	5640	17156.6	5951.9	2.4	5.2
Maharashtra	10162	3766	9883.7	4033.6	−2.8	6.6
Manipur	3701	1373	3822.5	1787.6	3.2	23.2
Meghalaya	3931	911	3819.2	1359.6	−2.9	33.0
Mizoram	2692	1171	2939.5	1351.6	8.4	13.4
Orissa	9377	3121	9135.4	3986.6	−2.6	21.7
Punjab	5440	2723	5442.9	2604.9	0.1	−4.5
Rajasthan	13754	4113	14433.5	4057.4	4.7	−1.4
Sikkim	1894	583	1819.5	647.3	−4.1	9.9
Tamil Nadu	5402	3628	5481.3	3730.3	1.5	2.7
Tripura	1196	425	1268.4	506.9	5.7	16.2
Uttar Pradesh	35641	8920	35730.9	8495.3	0.3	−5.0
Uttarakhand	5477	1283	5427.9	2086.7	−0.9	38.51
West Bengal	5240	2466	5549.3	2433.3	5.6	−1.3

### Consistency in Reporting Age at Marriage and Year of Birth

Consistency in responding to a question may also affect the quality of data. We check the consistency of data by examining the pattern of reporting age at marriage by current age of women and comparing with the expected and observed pattern. Current age here refers to the age at the time of the survey. Median age at marriage for female by the current age is presented in Table 7. The table gives the statistics for the cohorts 20–24 through 45–49 years. When progressing from older to younger cohorts the median age at marriage should either increase or remain constant. Declining median age or a ‘U’ shaped pattern suggests problems in the data [19]. From the table, we see that, the median age at marriage is declining from older to younger cohort in case of Sikkim, Mizoram, Kerala and Assam. Constant age at marriage is reported in the states of Punjab, Orissa, and Gujarat along with a ‘U’ shape curve for Goa. All these patterns suggest problems of age reporting. Two possible reasons may be identified for this discrepancy in age reporting, either the respondent is not able to recall the exact age at marriage or may intentionally increase the age at marriage to avoid reporting of marriages before the legally permitted age.

**Table 7:** Median age at marriage for women classified by age at the time of DLHS-3 survey for the major states of India.

State	Age at the time of survey					
	20–24	25–29	30–34	35–39	40–44	45–49
Andhra Pradesh	17	17	16	16	16	16
Arunachal Pradesh	19	19	18	19	19	20
Assam	18	19	19	19	19	20
Bihar	16	15	15	15	15	15
Chhattisgarh	18	17	16	16	16	16
Delhi	19	19	19	18	18	18
Goa	19	23	24	23	22	22
Gujarat	18	18	18	18	18	18
Haryana	18	18	18	17	17	17
Himachal Pradesh	20	20	20	19	19	19
Jammu & Kashmir	19	20	19	18	18	18
Jharkhand	17	17	17	17	17	18
Karnataka	17	18	17	17	17	17
Kerala	19	20	20	20	20	20
Madhya Pradesh	17	16	16	16	15	15
Maharashtra	18	18	17	17	17	17
Manipur	19	20	21	22	21	21
Meghalaya	18	19	19	20	20	21
Mizoram	19	20	20	20	20	21
Orissa	18	18	18	18	18	18
Punjab	20	20	20	20	20	20
Rajasthan	17	16	16	16	16	16
Sikkim	19	19	19	20	20	20
Tamil Nadu	19	19	19	19	18	18
Tripura	18	18	18	18	18	18
Uttar Pradesh	17	16	16	15	15	15
Uttarakhand	19	19	18	18	18	18
West Bengal	17	17	17	17	16	16

In addition to examining the median age at marriage, we also examined the percentage of births reported, in each year during the reference period prior to the survey to identify any specific pattern in reporting birth year of children. It may not be possible to measure the extent of displacement precisely, but examination of the year of birth distribution of children may help to identify the states where displacement is familiar. The results are presented in Table 8. Earlier studies suggest that older respondents may misplace their most recent births backward in time resulting in exaggeration of fertility in recent times [20]. In the absence of displacement of births, the distribution of births over the years is expected to remain more or less similar [21]. The pattern observed in the DLHS survey doesn't match the expected pattern. Some of the states show a gradual increase in the percentage of births while some other states show a decline in the percentage of births for the recent years. Some heaping is also noticed in reporting the year of birth. Among the major state, Punjab, Meghalaya, Delhi and Goa has almost equal percentage of birth in each year. Further, Jammu & Kashmir, Mizoram, Chhattisgarh and Himachal Pradesh reported equal percentage of births in the last three years. There was a gradual increase in the percentage of births for successive years in Assam and Uttarakhand. All these patterns of reporting the year of birth raise questions about the reliability of the responses in the survey.

**Table 8:** Percentage of births reported by ever married women for the four years prior to the DLHS-3 survey in the major states of India.

State	2004	2005	2006	2007
Andhra Pradesh	2.3	2.1	2.0	2.4
Arunachal Pradesh	1.2	1.3	1.2	0.9
Assam	2.9	4.0	4.3	4.3
Bihar	10.3	10.4	9.7	10.5
Chhattisgarh	3.1	2.7	2.7	2.7
Delhi	1.1	1.2	1.1	1.1
Goa	0.2	0.2	0.2	0.2
Gujarat	2.6	2.9	3.4	3.3
Haryana	3.6	3.5	5.9	3.4
Himachal Pradesh	0.9	1.0	1.0	1.0
Jammu & Kashmir	1.8	2.2	2.2	2.2
Jharkhand	4.8	5.0	4.6	4.9
Karnataka	3.9	3.5	3.2	3.6
Kerala	1.5	1.4	1.3	1.4
Madhya Pradesh	6.7	7.3	6.8	7.4
Maharashtra	4.8	4.4	4.5	4.4
Manipur	1.3	1.5	1.6	1.4
Meghalaya	1.3	1.3	1.3	1.2
Mizoram	1.3	1.2	1.2	1.2
Orissa	2.6	3.2	3.2	3.4
Punjab	2.4	2.4	2.5	2.4
Rajasthan	5.3	5.6	4.9	5.6
Sikkim	0.7	0.6	0.5	0.5
Tamil Nadu	3.4	3.0	2.9	2.9
Tripura	0.5	0.6	0.7	0.6
Uttar Pradesh	20.8	18.9	18.3	18.9
Uttarakhand	1.2	1.6	1.8	1.8
West Bengal	2.9	2.9	2.7	2.8

### Pattern of Skipping observed in the DLHS Survey

In a large scale survey, where the length of the questionnaire usually varies between 40–50 pages, it may be common tendency among respondents to skip some questions in order to shorten the duration of the survey. At times, this intentional skipping may affect the quality of data if the pattern of skip is same for respondents from different socio-economic background. As a result of intentional skipping, information on a large section of the questionnaire will be missing from the data. The skipping pattern observed in DLHS-3 survey is presented in Table 9. Interestingly more than half of the respondents in all the states reported no pregnancies since 2004. Proportion of women who reported no pregnancies since 2004 is as high as more than two third in Arunachal Pradesh and nearly two third in Tamil Nadu, Himachal Pradesh and Andhra Pradesh. Out of those women who reported getting pregnant since 2004; 45 percent each in Uttarakhand and Meghalaya and more than two fifth in Rajasthan, Jharkhand and Bihar reported that they had never received ANC during pregnancy.

**Table 9:** Percentage of skips observed during the DLHS-3 survey for the major states of India.

State	No pregnancies Since 2004	No ANC	Currently not pregnant	Haven't Heard of HIV	Haven't Heard of RTI
Andhra Pradesh	73.9	4.1	16.1	28.3	76.4
Arunachal Pradesh	76.6	37.2	27.4	27.9	80.0
Assam	63.1	25.7	12.9	46.1	83.2
Bihar	49.9	40.9	13.0	70.9	60.1
Chhattisgarh	62.4	20.6	13.2	62.2	61.4
Delhi	65.1	8.3	9.4	16.2	54.7
Goa	69.1	9.9	8.0	10.0	64.7
Gujarat	67.1	28.6	12.0	57.6	77.4
Haryana	63.6	12.2	13.5	35.3	60.9
Himachal Pradesh	72.8	13.4	12.7	18.1	55.2
Jammu & Kashmir	63.1	15.8	11.9	38.3	75.7
Jharkhand	55.4	44.3	12.4	74.9	82.8
Karnataka	69.2	9.9	14.6	17.1	62.9
Kerala	70.1	0.2	10.2	2.3	24.2
Madhya Pradesh	63.3	38.4	15.1	68.0	83.5
Maharashtra	67.2	8.3	14.6	29.9	72.8
Manipur	57.1	25.0	13.5	4.4	53.1
Meghalaya	51.8	44.5	18.7	51.7	92.3
Mizoram	58.8	10.5	21.1	7.3	55.2
Orissa	70.3	16.1	9.9	53.1	85.8
Punjab	70.3	16.9	10.7	13.1	40.9
Rajasthan	67.2	43.5	14.8	48.7	53.7
Sikkim	65.3	5.2	6.8	21.4	71.9
Tamil Nadu	71.9	1.1	11.5	8.6	73.1
Tripura	59.6	33.5	8.2	35.7	68.4
Uttar Pradesh	51.4	34.9	11.1	62.6	70.1
Uttarakhand	65.8	44.9	12.6	39.9	70.8
West Bengal	67.1	4.0	7.5	52.1	65.8

Nearly two third of respondents in Bihar, Uttar Pradesh, Chhattisgarh, Jharkhand and Madhya Pradesh reported that they never heard of HIV. Further, it is observed that more than half of the respondents in Gujarat, Meghalaya and West Bengal reported as not aware about HIV. When information was collected about the knowledge of RTI, more than 90 percent of the respondents in Meghalaya reported no knowledge on RTI. Other states, which follow Meghalaya are, Assam, Arunachal Pradesh, Jharkhand, Madhya Pradesh, Orissa, Andhra Pradesh, and Gujarat. High percentage of negative response to the questions related to reproductive and sexual health raises concern on the authenticity of the responses. These percentages may be an indication of the investigator bias while collecting data or it may also happen that people deliberately misreported these questions to escape answering the next section in the questionnaire.

The study also examined the skipping pattern by the background characteristics of the respondents, in order to identify if belonging to different socio-economic strata has any association with how they report to the issues on HIV and RTI. The pattern observed in responding to these sensitive issues is presented in Table 10. The results present clear difference knowledge of HIV for respondents from different socio-economic background. The percentage of respondents not aware of HIV is more than two times higher in rural areas than urban areas. The awareness of HIV and RTI is lacking among rural, poor and illiterate people. Although the level of awareness increases with the increase in living standard, but still approximately half of respondents in richest wealth quintile are not aware of RTI, which may lead to a question of reliability of the data.

**Table 10:** Percentage of Skips observed during the DLHS-3 survey by characteristics of respondent.

Background Characteristics	Not Aware of HIV	Not Aware of RTI
**Type of locality**		
Rural	50.5	71.9
Urban	20.6	57.0
**Religion**		
Hindu	42.7	67.4
Muslim	45.5	67.1
Christian	22.7	67.1
Sikh	15.2	42.4
Others	38.2	76.9
**Caste Group**		
Scheduled caste	45.5	70.3
Scheduled tribe	51.8	78.2
None of them/others	27.5	58.0
**Wealth Index Quintiles**		
Poorest	77.3	81.8
Second	64.9	77.7
Middle	46.1	74.0
Fourth	29.2	65.2
Richest	11.9	49.2
**Literacy**		
Literate	20.8	58.4
Illiterate	67.3	78.6
**Total**	**41.0**	**67.2**

## Discussion

In this paper, we examined three categories of errors in large scale surveys which are likely to affect the quality of data. We mainly examined the quality of age data with the help of the indices of digit preference and also by constructing some other indices to detect exclusion of women from the eligible age group. Further, inconsistency in response and intentional skipping has been examined through reporting of age at marriage, number of pregnancies, ANC visits, birth year of children and knowledge about HIV and RTI. In addition, number & time of visiting the household have also been examined to observe the association of fieldwork related factors with the quality of data.

It has come out from the results that digit preference in age reporting is present in the DLHS-3 data which needs to be taken care of before going for any further analysis on age related indicators. Age reporting during household interview of DLHS-3 has significance because women in the reproductive age group are screened out from the household survey to proceed with the ever-married and un-married women questionnaire. Some of the indicators are sensitive to changes in total population of that particular age group, in such cases even the slightest variations in reported ages may inflate the results. If women are deliberately pushed to some other age group than their actual ages, it will affect the estimation of indicators like age specific fertility rate, total fertility rate etc. which are based on the total population in the age group. If women are excluded from the lower (15–19) and upper (45–49) limit of reproductive age group, then information about some important vital events will be completely missing from the survey. Keeping in mind the volume of data collected and the geographical coverage of the survey, some amount of error is unavoidable. The extent of non-sampling error may be reduced if proper care is taken during data entry or through extensive training of the interviewer.

The most significant association of age reporting has been observed with the literacy of the respondent. Literacy of the individual at the micro level, and subsequently literacy at the state level both are reflected in the quality of age data. Earlier studies [5], [6] also agree that educated respondents provide a better response than uneducated respondents. Since age data is widely used in the estimation of different indicators, it is important that age of individuals is reported correctly. In addition to educational level; rural residence and low socio-economic status of respondent are also associated with age misreporting. Thus, it indicates that people from rural areas and belonging to low socio-economic status are also the most deprived in terms of educational attainment, and hence they are less likely to have any formal document as a proof for their actual age. So, the cumulative effect of being illiterate and belonging to a rural area and low socio-economic status lead to high age misreporting. Therefore, it may be said that education is important in order to obtain reliable answers in any survey. The study also observed that the incidence of digit preference is low among female than male. This finding is supported by an earlier study where the researchers observed low digit preference among female patients while recording their age [22]. The effect of digit preference may be reduced to some extent by grouping the data into five year age groups during analysis.

Another important finding of the study is that, in most of the states, female were excluded from the bordering age groups of reproductive span, which determines eligibility for the individual interview. Also, it results in deformed age structure and imbalance in sex ratio in the neighbouring age groups. This is a common occurrence in the age groups just outside the age range of eligibility for individual interview, but it is difficult to identify whether the exclusion is due to interviewer or respondent. This is important mainly due to the fact that, if women in the reproductive ages are misplaced from their actual age group then it will affect the results obtained from the survey in terms of estimating current fertility. An earlier study also pointed out that age heaping at the start of age groups, may affect the parity calculations [2]. After the findings related to age misreporting it is encouraging to find is that, the extent of age misreporting has declined in DLHS-3 compared to DLHS-2, in many states. The most significant changes have been observed in Rajasthan and Andhra Pradesh.

The study further observed inconsistency in reporting age at marriage, percentage of women pregnant at the time of the survey and birth year of children. These errors may affect estimates of current fertility and trends in fertility over time. People may have the tendency to inflate age at marriage in order to hide low age at marriage. Women may not wish to report current pregnancy in order to avoid questions on knowledge and use of contraception, intended or unintended pregnancy, care to be taken during pregnancy, interval between two births, preferred sex of the child, desired family size, or in some cases pregnancy may not be identified if it is in the very early stage. Actual reporting of birth year of children may be affected if the respondents are not able to place the births in the actual year, manipulate the birth years to maintain a minimum gap between two successive births, or they may deliberately misplace the births in order to skip answering questions related to immunization of young children. A little more probing by the investigator to relate the reported ages or dates with some important events in that year may help in obtaining more accurate information. Some of these errors may also arise due to lack of care in data entry. In addition to manipulating the age at marriage or birth year of children; more than half of the respondents in all the states reported no pregnancies since 2004. Looking at the proportion of births during the reference period and prevailing fertility rate in the country, there may be questions about reliability of the responses.

The study further observed under-reporting of reproductive and sexually transmitted diseases (HIV/RTI) in the survey. It may be the stigma attached to these issues that people refuse to discuss these diseases. People need to be sensitized about the importance of having proper knowledge about these sexually transmitted diseases and also spread the knowledge among the mass. Instead of asking direct questions about knowledge of HIV or RTI, it may be a better idea to collect information through some indirect questions. In terms of field work related factors, the results of the analysis points towards the fact that an increased number of visits to a household and selecting convenient time for conducting an interview may result in a better response rate and good quality data.

The study would like to conclude that age misreporting, inconsistent and incomplete response are the three important sources of error that are most likely to occur in a survey and also affect the survey results in many possible ways. Age misreporting may result in deformation of age structure and exclusion of women from individual interview. Inconsistency in reporting age at marriage and birth year of children will influence estimation of current and marital fertility and incomplete response to questions on HIV and RTI will give a wrong message about the level of awareness of these issues among the masses. In terms of the data from DLHS-3 survey, it is observed that age data shows preference for certain digits, but this error may be reduced to some extent if the data are grouped into five year age groups. In addition, reporting of ages for women in the reproductive age group suffers from in-transference and out transference from the actual age groups. In line with the conclusions of earlier studies with similar findings, it may be suggested that the upper age group for inclusion of women for individual interview may be extended to include women in 50–54 age group as well and then later do the analysis for women in the age group of 15–49 years [4]. In this study, DLHS-3 is used only to show how different errors may affect the quality of data. Since, the focus of this study was on age related data only and no other studies have attempted to examine the quality of DLHS-3 data, direct comparison of the results is also not possible. A comprehensive study is required to examine the quality of data for major policy related indicators so that these can be used properly for policy formulation.
